# Polymer Rheology and Processing of Nano- and Micro-Composites

**DOI:** 10.3390/ma15207297

**Published:** 2022-10-19

**Authors:** Ramón Pamies

**Affiliations:** Grupo de Ciencia de Materiales e Ingeniería Metalúrgica, Universidad Politécnica de Cartagena, Campus de la Muralla del Mar, 30202 Cartagena, Spain; ramon.pamies@upct.es

The development of new technologies strongly depends on the design of new materials. The combination of dissimilar materials opens a wide variety of opportunities to create composites with novel functions, properties, and applications [1]. Polymers are the most commonly used matrices due to their low density, simplicity of handling, low cost, and great potential in technological applications [2]. The addition of micro- and nano-particles not only provides effective mechanical and thermal stability but also offers new functionalities such as optical, electrical, electronic, or antiwear properties, among others. These properties mainly rely on the chemical nature, structure, and size of the fillers [3]. Therefore, the design of micro- and nano-composites is an increasingly important technology to improve the properties of polymeric materials with promising applications in different fields, such as nanotechnology [4], construction [5], aerospace [6], power energy [7], or biomedicine [8].

Rheology provides crucial knowledge regarding the interaction between the different phases added to polymeric matrices and the processing of composites. Thus, the rheological characterization and computational modeling of composites provide critical information about the compatibility of the different phases and the processing of the final material. Moreover, these novel materials demand the development of innovative processing methods and technologies that can result in novel applications.

One of the main challenges in the preparation of reinforced composites is obtaining uniformly dispersed fillers in the polymeric matrix. Fillers, especially in the case of nanomaterials, tend to agglomerate with a non-homogeneous distribution, which can act as defects [9]. This scenario is likely to promote stress concentration with the subsequent failure of the material [10].

In the case of nano-composites, the most popular techniques of fabrication include solvent processing, in situ polymerization, and melt blending [11], among others. Additive manufacturing, generally known as 3D printing, allows the fabrication of customized specimens with geometric complexity, one of the most widely used 3D printing techniques being Fused Deposition [12]. The manufacturing process can be a key factor in the final product. For example, Masarra et al. [13] studied the addition of graphene nanoplatelets to poly(lactic acid) (PLA) and polycaprolactone (PCL) biopolymers to fabricate electrically conductive nanocomposites. The results showed that the injection-molded samples were insulators, whereas the 3D-printed samples featuring the same graphene content were semiconductors.

Rheology is a powerful tool to develop new materials since it responds to the different conditions that occur in most of the above-mentioned manufacturing processes; for example, the flowing conditions during extrusion [14] or the viscoelasticity during interlayer welding in additive manufacturing [15]. Therefore, exploring the viscoelastic properties of these new materials is a fast and effective technique to predict processing conditions. There are extensive examples and applications of how rheological characterization helps in the design and fabrication of new composites: LDPE modified with layered double hydroxides with enhanced optical properties [16], PCL scaffolds with gelatin and nano-hydroxyapatite for biomedical applications [17], the addition of carbon nanophases on thermoplastic polymers [18], or the optimization of the extrusion of natural rubber/carbon black nanocomposites [19].

The addition of wood particles in recycled polyethylene increases the viscoelastic moduli due to poor compatibility and weak interfacial adhesion between the two phases, which is a promising result regarding common processing technologies such as extrusion or injection molding [20].

It was found that designing filaments of graphene-polyamide nanocomposites with steady shear viscosities of approximately 15% of the maximum printable viscosity for the desired printing conditions will be advantageous for easy material extrusion processing [21]. Carbon nanophases and other mineral fillers enhance the thermal properties of elastomeric materials. These micro- and nanocomposites exhibit an increase in flame-retardant behavior [22].

Self-healing composites exhibit interesting properties such as protection against corrosion. For example, the incorporation of an aminosilane into organic polymers using sol–gel chemistry has been evaluated through thermal, mechanical, and rheological properties [23]. Modified silica nanoparticles are also added to polyamides to modulate the crystallinity of the polymer, with an improvement in its rheological and mechanical properties [24].

Bionanocomposites are an emerging field of study. PLA is one of the most promising bioplastics to replace regular plastics. However, its mechanical, thermal, and barrier properties, among others, should be improved. The addition of nanoparticles by melt compounding is a widely used technique [25]. The rheological properties of the melt composites are of great importance to establish the processing conditions [26].

This Special Issue is intended to give an overview of the recent research dealing with the formulation of polymer-based nano- and micro-composites. These emerging materials have shown special properties and a wide range of applications. However, current manufacturing techniques face challenging difficulties to process these materials. The rheological characterization of composites gives critical information for their design, modeling, and fabrication.

## References

[1] Moya J.S., Lopez-Esteban S., Pecharromán C. (2007). The challenge of ceramic/metal microcomposites and nanocomposites. Prog. Mater. Sci..

[2] Nawaz H., Umar M., Maryam R., Nawaz I., Razzaq H., Malik T., Liu X. (2022). Polymer Nanocomposites based on TiO_2_ as a reinforcing agent: An Overview. Adv. Eng. Mater..

[3] Zhao B., Zhu L. (2009). Mixed Polymer Brush-Grafted Particles: A New Class of Environmentally Responsive Nanostructured Materials. Macromolecules.

[4] Zhong M., Zhang M., Li X. (2022). Carbon nanomaterials and their composites for supercapacitors. Carbon Energy.

[5] Rupal A., Meda S.R., Gupta A., Tank I., Kapoor A., Sharma S.K., Sathish T., Murugan P. (2022). Utilization of Polymer Composite for Development of Sustainable Construction Material. Adv. Mater. Sci. Eng..

[6] Rawal S., Sidpara A.M., Paul J. (2022). A review on micro machining of polymer composites. J. Manuf. Process..

[7] Knych T., Mamala A., Kwaśniewski P., Kiesiewicz G., Smyrak B., Gniełczyk M., Kawecki A., Korzeń K., Sieja-Smaga E. (2022). New Graphene Composites for Power Engineering. Materials.

[8] Kasi G., Gnanasekar S., Zhang K., Kang E.T., Xu L.Q. (2022). Polyurethane-based composites with promising antibacterial properties. J. Appl. Polym. Sci..

[9] Tätte T., Hussainov M., Amiri M., Vanetsev A., Paalo M., Hussainova I. (2022). Rheological Properties of MWCNT-Doped Titanium-Oxo-Alkoxide Gel Materials for Fiber Drawing. Materials.

[10] Cho J., Boccaccini A.R., Shaffer M.S.P. (2009). Ceramic matrix composites containing carbon nanotubes. J. Mater. Sci..

[11] Sanes J., Sánchez C., Pamies R., Avilés M.-D., Bermúdez M.-D. (2020). Extrusion of Polymer Nanocomposites with Graphene and Graphene Derivative Nanofillers: An Overview of Recent Developments. Materials.

[12] Calafel I., Aguirresarobe R.H., Peñas M.I., Santamaria A., Tierno M., Conde J.I., Pascual B. (2020). Searching for Rheological Conditions for FFF 3D Printing with PVC Based Flexible Compounds. Materials.

[13] Masarra N.-A., Batistella M., Quantin J.-C., Regazzi A., Pucci M.F., El Hage R., Lopez-Cuesta J.-M. (2022). Fabrication of PLA/PCL/Graphene Nanoplatelet (GNP) Electrically Conductive Circuit Using the Fused Filament Fabrication (FFF) 3D Printing Technique. Materials.

[14] Ji H.S., Park G., Jung H.W. (2022). Rheological Properties and Melt Spinning Application of Controlled-Rheology Polypropylenes via Pilot-Scale Reactive Extrusion. Polymers.

[15] Mackay M.E. (2018). The importance of rheological behavior in the additive manufacturing technique material extrusion. J. Rheol..

[16] Monzó F., Caparrós A.V., Pérez-Pérez D., Arribas A., Pamies R. (2019). Synthesis and Characterization of New Layered Double Hydroxide-Polyolefin Film Nanocomposites with Special Optical Properties. Materials.

[17] Wang C., Liu J., Lin M., Zhang R., Li Y., Li Y., Zou Q. (2022). Extrusion deposition 3D printed PCL/gel/n-HA composite scaffold for bone regeneration. Int. J. Polym. Mater. Polym. Biomater..

[18] Sanes J., Ojados G., Pamies R., Bermudez M.D. (2019). PMMA nanocomposites with graphene oxide hybrid nanofillers. Express Polym. Lett..

[19] Wu H., Lu X., He Y., Qu J.-P. (2022). Continuous mixing process and properties of NR/CB nanocomposites based on elongational rheology. Compos. Part B Eng..

[20] Patti A., Cicala G., Acierno S. (2021). Rotational Rheology of Wood Flour Composites Based on Recycled Polyethylene. Polymers.

[21] Lee K., Brandt M., Shanks R., Daver F. (2020). Rheology and 3D Printability of Percolated Graphene–Polyamide-6 Composites. Polymers.

[22] Rybiński P., Syrek B., Marzec A., Szadkowski B., Kuśmierek M., Śliwka-Kaszyńska M., Mirkhodjaev U.Z. (2021). Effects of Basalt and Carbon Fillers on Fire Hazard, Thermal, and Mechanical Properties of EPDM Rubber Composites. Materials.

[23] Lollivier G., Gressier M., Ansart F., Aufray M., Menu M.-J. (2021). Influence of Hybrid Sol-Gel Crosslinker on Self-Healing Properties for Multifunctional Coatings. Materials.

[24] Jeziorska R., Szadkowska A., Studzinski M. (2022). Morphology and Properties of Poly(2,6-dimethyl-1,4-phenylene oxide)/Polyamide 11 Hybrid Nanocomposites: Effect of Silica Surface Modification. Materials.

[25] Murariu M., Galluzzi A., Paint Y., Murariu O., Raquez J.-M., Polichetti M., Dubois P. (2021). Pathways to Green Perspectives: Production and Characterization of Polylactide (PLA) Nanocomposites Filled with Superparamagnetic Magnetite Nanoparticles. Materials.

[26] Sánchez-Rodríguez C., Avilés M.-D., Pamies R., Carrión-Vilches F.-J., Sanes J., Bermúdez M.-D. (2021). Extruded PLA Nanocomposites Modified by Graphene Oxide and Ionic Liquid. Polymers.

